# First Analysis of Mild Behavioral Impairment in a Sample of Mexican Older Adults

**DOI:** 10.3390/neurosci7020036

**Published:** 2026-03-13

**Authors:** Ángela Acosta-Amaya, Salvador Sánchez-Badajos, David J. Dávila-Ortiz de Montellano, Alberto Ortega-Vázquez, Ramiro Ruiz-Garcia, Nancy Monroy-Jaramillo, Yaneth Rodríguez-Agudelo

**Affiliations:** 1 Laboratorio de Neuropsicología Clínica, Instituto Nacional de Neurología y Neurocirugía, Manuel Velasco Suárez (INNNMVS), Mexico City 14269, Mexico; angela_patricia_acosta@psicologia.unam.mx; 2 Facultad de Psicología, Universidad Nacional Autónoma de México (UNAM), Mexico City 04510, Mexico; 3 Doctorado en Ciencias Biológicas y de la Salud, Universidad Autónoma Metropolitana (UAM), Mexico City 04960, Mexico; 2233800589@alumnos.xoc.uam.mx; 4 Departamento de Genética, Instituto Nacional de Neurología y Neurocirugía, Manuel Velasco Suárez (INNNMVS), Mexico City 14269, Mexico; david.montellano@innn.edu.mx; 5 Departamento de Sistemas Biológicos, Universidad Autónoma Metropolitana (UAM) Unidad Xochimilco, Mexico City 04960, Mexico; aortega@correo.xoc.uam.mx; 6 Departamento de Neuropsiquiatría, Instituto Nacional de Neurología y Neurocirugía, Manuel Velasco Suárez (INNNMVS), Mexico City 14269, Mexico; ramiro.ruiz@innn.edu.mx

**Keywords:** mild behavioral impairment, *APOE* gene, subjective cognitive decline, mild cognitive impairment, normal cognition

## Abstract

Mild behavioral impairment (MBI) constitutes a late-life transition state that is associated with an increased risk of cognitive impairment and dementia. Herein, we cross-sectionally describe the MBI construct and its relationship with cognitive status in Mexican-Mestizos (MM) older adults. Participants were classified according to their cognitive and behavioral statuses using tests administered to older adults and their informants. *APOE*_rs429358/rs7412 variants were genotyped by real-time PCR. Multivariate correlation and Principal Components Analysis (PCA) were used in statistical analysis. A total of 246 participants were included, 56.1% were classified as individuals with NC, 13.0% had subjective cognitive decline, and 30.9% had mild cognitive impairment. A total of 37% (91/246) of participants from all over the cognitive spectrum met the MBI criteria; among this group, *APOEε4* homozygosity was associated with two subdomains of the MBI. Subjective cognitive complaint, symptoms of depression, and cognitive decline reported by the informant were associated with an increased risk for MBI (ORs in the range of 4.7–15.89). The first three components of PCA explained 68.0% of the variance of the dataset, including the MBI-checklist total score as a main contributor. Well-known risk factors for dementia also correlated with this PCA. MBI could be a potential marker for cognitive decline in non-demented MM elderly people; however, observed associations should be confirmed in future longitudinal studies.

## 1. Introduction

Research on neurodegenerative disorders, including Alzheimer’s disease (AD), has focused on early detection through clinical markers in pre-dementia states [1,2,3]. In 2024, the first Mexican survey regarding early diagnosis of dementia in primary care revealed that only 17% of patients received early diagnosis of dementia in primary care centers in the Mexican public health sector. This emphasizes the need to boost early detection rates to ensure the timely referral of patients with dementia to specialized centers [4].

In pre-dementia stages, neuropsychiatric symptoms (NPS) are highly prevalent and have a strong relationship with AD biomarkers, particularly those related to beta-amyloid pathology, across different stages of the cognitive continuum [5,6,7,8]. It is imperative to recognize the role of NPS in the diagnostic criteria for neurodegenerative diseases; its integration in research and clinical practice allows for more accurate detection and facilitates the comprehension of the biological substrate and its relationship with other variables, such as the cognitive component [1]. The International Society to Advance Alzheimer’s Research and Treatment (ISTAART) introduced mild behavioral impairment (MBI) as a diagnostic construct for pre-dementia stages, given its potential for early detection of individuals at risk for developing dementia, and its predictive value for AD [2]. MBI can appear at all stages of the cognitive continuum, i.e., in individuals with normal cognition (NC), subjective cognitive decline (SCD), or mild cognitive impairment (MCI) [2,9]. The Mild Behavioral Impairment-Checklist (MBI-C) operationalizes the concept of MBI from ISTAART, measures a selected list of NPS, and can aid in predicting the risk for AD and other neurodegenerative diseases [10,11].

A longitudinal study in 1273 participants with NC or MCI found that those meeting MBI criteria had faster progression to dementia than non-MBI NPS (i.e., NPS that are transient or attributable to psychiatric conditions). Participants with MBI showed a broader pattern of neurodegeneration and additional associations with AD structural biomarkers of interest, including lower bilateral hippocampal volume and cortical thickness in the AD meta-region, with the exception of the entorhinal cortex for participants with NC, whereas non-MBI NPS did not [12]. Therefore, MBI could be considered as a diagnostic entity that improves the prognostic accuracy of neurodegenerative diseases in individuals with NC and MCI [12,13].

MBI-C includes five domains: decreased motivation, affective and emotional dysregulation, impulse dyscontrol, social inappropriateness, and abnormal perception or thought content manifesting as delusions and/or hallucinations [2,10,14]. The most frequently reported domains are affective and emotional dysregulation or impulse dyscontrol, followed by decreased motivation in samples of individuals over 50 years of age [15,16,17].

The overall prevalence of MBI in non-demented older adults ≥55 years of age has been documented between 33.5 and 37.2%, and this prevalence range varies according to the population studied, recruitment methods, settings, and the specific evaluation scales used [16,17,18]. However, the stratification of individuals by cognitive status shows the following prevalence in MBI: 16.1–27.6% in individuals with NC, 35.8–43.1% in SCD, and 45.5–52.7% in MCI [17,18,19]. MBI is associated with lower cognitive performance, cognitive decline, and an increased risk of progression to dementia [6,18,19,20,21,22,23,24]. The domains of affective and emotional dysregulation and impulse dyscontrol are the most predictive of future diagnoses [11].

The gene encoding for apolipoprotein E (APOE) is known as the most important modulator of AD risk, and the inheritance of the ε4 allele of *APOE* significantly increases the AD risk [25]. In the Mexican-Mestizo population (MM), the *APOEε4* carrier status increases the risk of cognitive decline by up to two times [26]. *APOEε4* status has been associated with affective and emotional dysregulation [22,27,28], but no evidence has been found of its association with other subdomains or severity of MBI [23,27]. It has been suggested that *APOEε4* status could mediate the relationship between MBI and cognitive decline [22,27,28].

MBI could be conceptualized as a mediator of cognitive decline or a product of multifactorial risk, i.e., depression, diabetes, and vascular conditions associated with AD biomarkers [22,29]. Therefore, this highlights the need for a biopsychosocial and multifaceted approach to detect, manage, and prevent MBI [22,29,30]. It is plausible that MBI can serve as an early marker or prodromal state of AD, given the strong evidence linking MBI to cognitive decline, altered brain function, neuropathological alterations, and genetic etiology [6,11,12,31]; consequently, the MBI is considered the neurobehavioral axis of pre-dementia risk states, and complementary to the traditional neurocognitive axis [6,9,11,24]. In addition, the MBI has been used as a classification strategy for older adults without dementia to identify a group at higher risk for AD [31]. The working group of the Clinical Trials on Alzheimer’s Disease Conference (CTAD) advises integrating MBI into the assessment and management of NPS; they also provide recommendations for an MBI assessment in AD prevention trials [3]. However, the reports about MBI in Latin American populations are scarce. We aimed to characterize MBI in MM older adults and explore its potential associations with cognitive, clinical, and sociodemographic parameters, along with the *APOE* genotype. Their contribution to the dimensionality of the dataset is relevant to describing the cognitive health of MM older adults.

## 2. Materials and Methods

Mexican older adults aged ≥60 years who have finished at least the elementary education level and who have no dementia and no history of traumatic brain injury, neurological disease, or psychiatric illness except for mild or moderate depression and anxiety were enrolled in this study. In addition, each participant was required to have an informant, defined as a family member or partner with ≥5 years of relationship with the older adult. For blood sampling and the analysis of *APOE* genotype, participants were required to be MM, which was defined as individuals descending, on both maternal and paternal lines, from parents and grandparents born in Mexico

**Figure 1:**
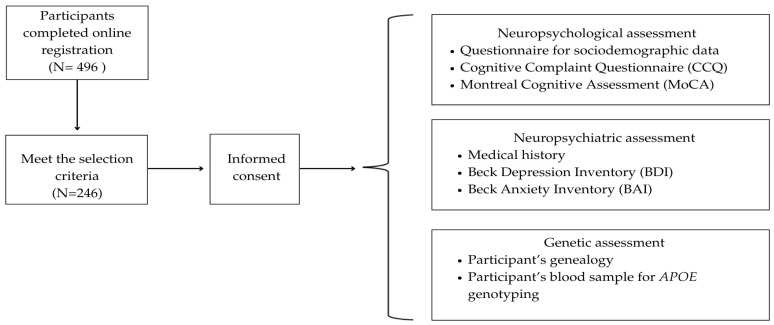
Flowchart illustrating the procedures and assessments carried out on the participants. N corresponds to the number of participants included in each step. Some of them were excluded from the study for not meeting the inclusion criteria (relationship with another participant, foreign background, or because they had psychiatric symptoms other than mild-to-moderate depression or anxiety), or the participant could not be invited to the study because it was not possible to contact him/her by phone.

A cross-sectional, descriptive, and correlational study was conducted. Participants were invited through the institution’s social media channels and through visits to cultural centers for elderly people at the Instituto Nacional de las Personas Adultas Mayores (INAPAM) in Mexico City. The procedure and evaluations were conducted in a single in-person session lasting ≈ 120 min, as described in Figure 1. Two clinical neuropsychologists conducted telephone interviews with informants using the Informant Questionnaire on Cognitive Decline (IQCODE) and the Mild Behavioral Impairment Checklist (MBI-C) [10,32]. The research protocol was approved by the Research and Ethics Committees of the Instituto Nacional de Neurología y Neurocirugía (INNN_139/2023). This study followed the ethics principles contained in the Declaration of Helsinki for research with human participants.

### 2.1. Instruments

*Questionnaire for Sociodemographic data and Medical History*. This instrument contains items to collect sociodemographic and medical history information.

*Cognitive Complaint Questionnaire (CCQ)*. This questionnaire explores cognitive complaints in older adults in the domains of attention, orientation, memory, language, executive functions, praxis, and gnosis. It consists of 24 items, 4 per cognitive domain, and its response format is a Likert scale, namely: 0, never; 1, almost never; 2, sometimes; 3, frequently; and 4, always. A cut-off point of 22 is considered for a significant subjective cognitive complaint [33].

*Montreal Cognitive Assessment (MoCA)*. It is a screening tool for cognitive function among older adults that assesses the domains of verbal episodic memory, visuospatial ability, executive function, attention/working memory, language, and orientation [34,35]. In this study, a score of 25 was used as a cut-off point for cognitive impairment, with a sensitivity ranging from 74 to 83.8% and a specificity ranging from 70 to 70.8% [36,37]. This cut-off value has also been adjusted for years of education and age group in an adult MM population [38].

*The Beck Depression Inventory (BDI).* BDI measures the severity of somatic and cognitive symptoms of depression. It consists of 21 Likert-scale items; 0 represents the absence of symptoms, and 3 represents high symptom frequency and severity. For mild depression, the cut-off point is 10 [39].

*Beck Anxiety Inventory (BAI)*. This inventory consists of 21 Likert-based items, where 0 indicates minimal or no anxiety symptom level; 1 = mild anxiety; 2 = moderate anxiety; and 3 = severe anxiety, with a mild level of anxiety considered to be 6 points or higher [40].

*Informant Questionnaire on Cognitive Decline in the Elderly (IQCODE)*. This questionnaire explores the informant’s report on the changes in the older adults over the past five years regarding performance in areas requiring memory skills and other cognitive domains. This is a 26-item questionnaire with a Likert-based response option: 1, much improved; 2, a bit improved; 3, not much change; 4, a bit worse; and 5, much worse [41]. There is a Spanish version that was validated, and 87 is the cut-off point for MCI [32].

*The Mild Behavioral Impairment Checklist (MBI-C)*. MBI-C operationalizes the MBI ISTAART criteria for its measurement and considers the symptoms of the last 6 months among the participants [2]. It is a 34-item scale, and its structure is directly aligned with the five domains of the MBI criteria. The scoring system consists of indicating the severity of the behavioral change assessed by the item (0 = absent; 1 = mild; 2 = moderate; and 3 = severe); these are added together to generate a score for each domain and a total score (ranging from 0 to 102) [10]. Herein, the version of the MBI-C for the Spanish population was used [42], and the cut-off point for MBI was set at 6, as reported elsewhere [43,44].

### 2.2. Classification of Cognitive Status

The criteria for determining the cognitive status were as follows: NC: participants with normal cognitive performance (MoCA ≥ 2 5) and absence of significant cognitive complaint (CCQ < 22 and/or IQCODE 87/88); SCD: significant subjective cognitive complaint (CCQ ≥ 22) and normal cognitive performance (MoCA ≥ 25) [45,46,47]; and MCI: significant cognitive complaint either subjective (CCQ ≥ 22) and/or reported by the informant (IQCODE ≥ 87/88), and low cognitive performance (MoCA < 25) [48,49]. For the multivariate analysis, the cognitive status was considered as an ordinal variable, as follows: 1 = NC, 2 = SCD, and 3 = MCI.

### 2.3. Genetic Analysis

DNA was extracted from peripheral blood samples using a commercial kit in an automated QIAcube (QIAGEN, Hilden, Germany) system. The qualitative and quantitative analysis of genomic DNA was assessed by agarose gel electrophoresis and using the NanoDrop™ spectrophotometer (Thermofisher, Waltham, MA, USA), respectively. The single-nucleotide variants (SNVs) rs7412 and rs429358 (c.604C > T: p.Arg176Cys and c.466T > C: p.Cys130Arg, respectively) of *APOE* were genotyped by real-time PCR (polymerase chain reaction) using a QuantStudio Real-Time PCR system (Thermofisher, Waltham, MA, USA) and allelic discrimination. Commercial TaqMan probes (Thermofisher, Waltham, MA, USA) were used to discriminate *ε2*, *ε3*, and *ε4* alleles; reference DNA samples of known *APOE* genotype (confirmed by direct sequencing) and a non-template control were included in each run to validate the method. Then, to facilitate the analysis, the samples with *ε2*/*ε2* and *ε2*/*ε3* genotypes were classified as allelic group *ε2*, the *ε3*/*ε3* genotype as allelic group *ε3*, and the *ε3*/*ε4* and *ε4*/*ε4* genotypes as allelic group *ε4*. *APOEε4* carrier status was classified as absent, heterozygous, and homozygous (*ε4*/*ε4*) (i.e., 0, 1, or 2 copies of *ε4* allele, respectively).

### 2.4. Statistical Analysis

Frequencies and dispersion measures were used for descriptive statistics. The normality of the variables was assessed by the Kolmogorov–Smirnov test. To identify differences between groups based on sociodemographic, clinical, and genetic variants, different tests were used, as appropriate, including the chi-square test, Fisher’s exact test, and the Mann–Whitney U or Kruskal–Wallis test. To determine the Hardy–Weinberg (HW) equilibrium, expected genotype frequencies were calculated from observed allele frequencies. The chi-square (χ^2^) test was used to compare observed vs. expected genotype frequencies; if this test shows a *p*-value > 0.05, then the population is considered to be in HW equilibrium.

Odds ratios (ORs) with 95% confidence intervals (CI_95%_) were calculated to identify associations between variables of interest and the presence of MBI. A *p*-value less than 0.05 was considered statistically significant. Multivariate correlation analysis was used to establish the magnitude of the association between the variables identified as risk factors for MBI. Additionally, to reduce the high-dimensional data, Principal Component Analysis (PCA) was carried out. This analysis allowed us to identify the variables with the highest contribution to the data variance. The scores were transformed into Z-scores to obtain standardized data. The Kaiser–Meyer–Olkin (KMO) measure of sampling adequacy and Bartlett’s test of sphericity were performed [50]. Only components with eigenvalues >1 were considered. A scree plot was considered to determine the number of principal components (PC) to be extracted in the PCA. Each PC explains a percentage of the total variance in the data. Then, PCA biplots (PC1+PC2) were generated to visualize distribution and variable contributions applied to cognitive status and to MBI. Statistical analysis of the data was carried out with the Statistical Package for the Social Sciences (SPSS) version 27 and R version 4.5.1 [51].

## 3. Results

### 3.1. Sociodemographic, Cognitive, Clinical, and Genetic Characteristics of the Sample

Four hundred and ninety-six participants were originally recruited; however, 250 of them did not meet the inclusion criteria (e.g., relationship with another participant, foreign background, or because they had psychiatric symptoms other than mild-to-moderate depression or anxiety), or the participant could not be invited to the study because it was not possible to contact him/her by phone (Figure 1). Therefore, the studied sample included 246 MM older adults, and the mean age was 69.88 ± 6.58 years and they reported having 13.56 ± 3.57 years of education. Herein, convenience sampling was used, with a higher participation rate among women (N = 205), representing 83.3% of the cohort. The MoCA mean score was 25.06 ± 3.14 and the levels of cognitive status of participants were distributed as follows: 56.1% (N = 138) were classified as individuals with NC, 13.0% (N = 32) as SCD, and 30.9% (N = 76) as MCI (Table 1).

Participants with NC had significantly lower BDI and BAI scores (*p* < 0.001) and a higher *APOEε3* allele frequency (*p* > 0.05) compared to the SCD and MCI groups. The frequency of the *APOEε4* allele was higher in the SCD group than in the MCI and NC groups (*p* = 0.018). The group with MCI had fewer years of education and the lowest MoCA scores compared to the other groups (*p* < 0.05). IQCODE and CCQ scores were similar between the SCD and MCI groups, but significantly different from the group with NC (Table 1).

Regarding the allelic frequency of *APOE*, 87.40%, 2.03%, and 10.57% of the samples were carriers of *APOEε3*, *APOEε2*, and *APOEε4* alleles, respectively (Table 1). The distribution of the genotypes was as follows: *APOEε3/ε3* (N = 190, 77.2%), *APOEε3/ε4* (N = 43, 17.5%), *APOEε2/ε3* (N = 8, 3.3%), *APOEε4/ε4* (N = 4, 1.6%), and one participant showed *APOEε2*/*ε4* genotype (0.4%), and the genotype *APOEε2/ε2* was not observed. The relative frequencies of *APOE* were in the range of equilibrium of Hardy–Weinberg (*p* > 0.05) and were similar to the previously reported MM population values [52,53,54].

### 3.2. MBI Characteristics and Its Frequency in the Cohort

The frequency of each MBI domain reported in the entire cohort (N = 246) in descending order was: 59.3% (N = 146) for impulse dyscontrol, 49.2% (N = 121) for affective and emotional dysregulation, 38.6% (N = 95) for decreased motivation, 26.0% (N = 64) for social inappropriateness, and 12.2% (N = 30) for abnormal perception/thought content. Scoring of total and particular domains of MBI-C was similar between the SCD and MCI groups and higher than that of individuals with NC (Table S1).

### 3.3. Sociodemographic and Clinical Characteristics and APOEε4 Status Between Participants with and Without MBI

A total of 37.0% (91/246) of participants met criteria for MBI (cut-off point ≥6.0 in MBI-C); 26.1% (N = 36) of them had NC, while 59.4% (N = 19) and 47.4% (N = 36) were considered in the groups of SCD and MCI, respectively. The frequency of MBI was higher in SCD vs. MCI group, but this was nonsignificant (*p* = 0.256). From highest to lowest, the reported frequency of the five MBI-C domains within the group of participants with MBI was: impulse dyscontrol (95.6%), affective and emotional dysregulation (89.0%), decreased motivation (84.6%), social inappropriateness (58.2%), and abnormal perception/thought content (31.9%). The group with MBI presented higher levels of cognitive complaints and a higher frequency of depression and anxiety symptoms (*p* < 0.001) than the non-MBI group. Participants with MBI were younger (68.48 ± 6.42 vs. 70.70 ± 6.60 years, *p* = 0.009) and showed higher percentages of depression (53.8% vs. 32.9%, *p* = 0.002) and anxiety history (36.3% vs. 23.9%, *p* = 0.041) than the non-MBI group. In contrast, sex, years of education, marital status, cognitive performance (MoCA), and *APOE* allele distribution were similar in both groups (Table 2). Sociodemographic and clinical characteristics, as well as *APOEε4* status, were compared between female and male participants. This analysis revealed differences in years of education, marital status, and clinical antecedents of depression or hypertension, while MBI characteristics were similar (Table S2).

### 3.4. Comparison of MBI-C Domain Scores According to the Participants’ APOEε4 Carrier Status

**Figure 2:**
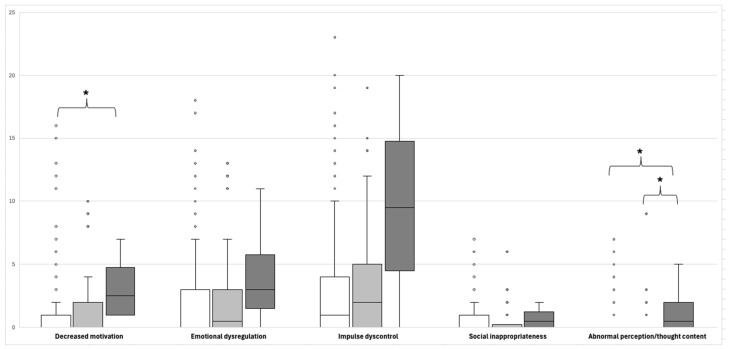
Boxplots showing the score for each domain of the Mild Behavioral Impairment-Checklist according to the *APOEε4* carrier status of participants. Each bar corresponds to the *APOEε4* carrier status: absence (white), one allele (light gray), or two *APOEε4* alleles (dark gray), respectively. * *p* < 0.05.

The observed *APOEε4* allele frequency in this study was like that reported in prior MM studies (*p* > 0.05) (Table S3). Regarding total or individual domains of MBI-C scoring, no differences were found between *APOEε4* carriers and non-carriers (Table 3). Then, the comparison of MBI-C domain scores by number of *APOEε4* alleles (i.e., absence vs. heterozygous vs. homozygous or 0–1–2 alleles) demonstrated differences in the domains of decreased motivation (0 vs. 2 alleles, *p* = 0.013) and abnormal perception or thought content (0 vs. 2 alleles, *p* = 0.027, and 1 vs. 2 alleles, *p* = 0.021) domains (Table 3 and Figure 2). Similar results were obtained in the association analysis of the *APOEε4* allele with the domains of the MBI-C in dominant and recessive models (Table S4).

### 3.5. Risk Association and Correlation Analysis

**Figure 3:**
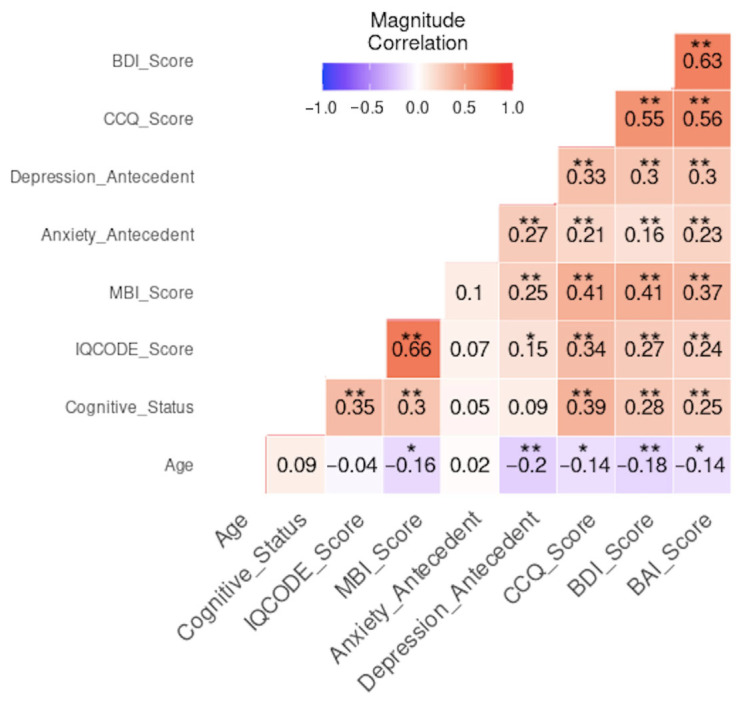
Multivariate correlation analysis of age, antecedents of depression or anxiety, and psychological variables evaluated in the participants. Pearson’s correlation was used. * *p* < 0.05, ** *p* < 0.01. IQCODE, Informant Questionnaire on Cognitive Decline in the Elderly; MBI, mild behavioral impairment; CCQ, Cognitive Complaint Questionnaire; BDI, Beck Depression Inventory; BAI, Beck Anxiety Inventory. Cognitive status was coded as follows: 1 = NC, 2 = SCD, and 3 = MCI.

To explore the association between all the variables and the presence of MBI, ORs were calculated only for statistically significant variables. This analysis revealed that SCD and MCI groups, scores for cognitive complaints (IQCODE and CCQ), depression, or anxiety (BDI, BAI), as well as a history of depression or anxiety, were associated with an increased risk for MBI (Table 4). On the contrary, NC and being older than 70 years would seem to have a decreased likelihood of developing MBI. The multivariate analysis showed correlations between MBI-C total score and IQCODE (r = 0.66, *p* < 0.01), CCQ (r = 0.41, *p* < 0.01), and BDI (r = 0.41, *p* < 0.01) (Figure 3).

### 3.6. Principal Component Analysis (PCA)

**Figure 4:**
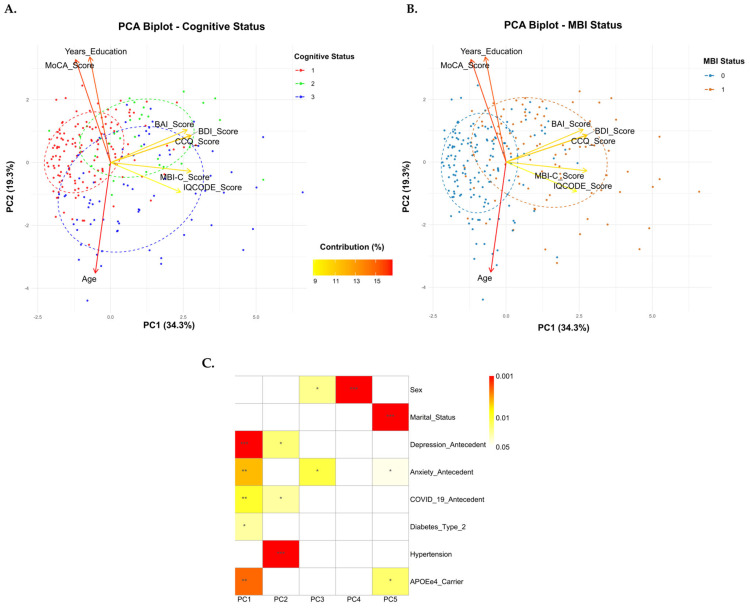
Principal Component Analysis (PCA) and its correlation with covariables. (**A**) Biplot applied to cognitive status, coded as follows: 1 = normal cognition (red), 2 = subjective cognitive decline (green), and 3 = mild cognitive impairment (blue). (**B**) Biplot applied to MBI status, coded as follows: 0 = absent MBI (blue) and 1 = present MBI (orange). Colored arrows indicate the percentage of contribution of the variables, and the code for this color gradient is depicted in the horizontal bar at the center of the Figure. PC1 and PC2 are principal components. (**C**) Heatmap of the correlation analysis of PCA vs. covariables. Five PCs are shown. The color gradient bar on the right indicates the magnitude of the correlation, and its significance is symbolized by asterisks: * *p* < 0.05, ** *p* < 0.01, and *** *p* <0.001. BDI, Beck Depression Inventory; BAI, Beck Anxiety Inventory; CCQ, Cognitive Complaint Questionnaire; IQCODE, Informant Questionnaire on Cognitive Decline in the Elderly; MBI-C, Mild Behavioral Impairment-Checklist; MoCA, Montreal Cognitive Assessment.

PCA was used to explore the relationship of the included variables and their contribution to the variance of the dataset. The KMO measure was 0.69, and Bartlett’s test was significant (*χ*^2^(28) = 494.933, *p* < 0.001), confirming the appropriateness of the data for PCA. The PCA revealed three components (PC1, PC2, and PC3) with eigenvalues greater than 1.0, explaining 68.0% of the total variance of data. The PC1 explained 34.3% of the variance, and the variables with the highest load for this dimension were CCQ, BDI, MBI-C, and BAI. The contribution of PC2 to the total variance of the data was 19.3%. The variables with the greatest contribution to PC3 (14.4% of the contribution to the total variance) were IQCODE, BAI/BDI, MBI-C, sex, and anxiety history (*p* < 0.05) (Table 5). A 2D biplot of the first two principal components was preferred because PC1 and PC2 together captured the majority of the data variance (53.6%) by using the loading vectors and PC scores, making them sufficient for visualizing the data’s essential structure (Figure 4). Participants with NC clustered around PC2, while the group with SCD was distributed between dimensions PC1 and PC2. The group with MCI showed a more dispersed distribution in the PCA biplot (PC1+PC2) of cognitive status (Figure 4A). Participants with MBI tended to cluster around PC1, whereas the participants without MBI were grouped in a scattered manner around PC2 (Figure 4B) in the PCA biplot applied to MBI. The correlation of PCA with other covariates showed that depression or anxiety history, *APOEε4* status, COVID-19, and diabetes were significantly correlated with PC1 (*p* < 0.05) (Figure 4C). The variables that contributed to the greatest variance in PC2 were age, years of education, MoCA, hypertension, and antecedents of COVID-19 (*p* < 0.05). In addition, depression history also correlated with PC2 (*p* < 0.05) (Figure 4C).

## 4. Discussion

There is scarce research regarding MBI within Latin Americans, including the Mexican population. To our knowledge, this is the first integrative study addressing sociodemographic, clinical, and cognitive characteristics along with *APOE* genotype and their association with MBI in MM older adults.

A meta-analysis has reported that the prevalence of MBI is 17.0% in individuals with NC, 35.8% in SCD, and 45.5% in MCI [18]. The frequency of MBI in our sample was 37.0%, which agrees with what has been documented in other populations (33.5–37.2%) [16,17,18]. Our participants with NC showed the lowest frequency of MBI (26.1%), followed by the group with MCI (57.4%) and by the group with SCD (59.4%). Participants with NC vs. MCI or SCD showed differences in MBI-C, while participants with SCD or MCI exhibited a similar frequency of MBI (*p* = 0.256), which is in accordance with a prior study carried out in a memory clinic [55]. In the same line, there is a study performed in a community-based Southeast Asian cohort in which the authors did observe differences between participants with MCI and NC; however, they did not find differences in total or any domain scores of MBI-C between participants with SCD or MCI, suggesting that behavioral symptoms emerge before cognitive ones [56]. One possible explanation for the similar MBI frequencies observed between the SCD and MCI groups could be that the participants with SCD came to this study because of concerns about their cognitive health, as reported elsewhere [55], as opposed to what might have been found in a randomly selected sample or population study.

The most frequently reported domains of MBI-C in the entire cohort (N = 246) and in the group with MBI were impulse dyscontrol (59.3% and 95.6%, respectively), preceded by affective and emotional dysregulation (49.2% and 89.0%, respectively). These data are in agreement with what has been previously documented [11,17,57]. There are some reports with differences in the frequencies of the five domains of the MBI-C, but this may be influenced by the age of participants, including [15] the type of relationship between the informant and the older adult, cultural beliefs, educational level, and other factors that contribute to the informant’s denial/minimization [17]. MBI is often studied in people aged 50 or older; our research design choice only included participants ≥60 years. In this context, considering this deviation from the commonly used age threshold in MBI criteria, our prevalence findings should be interpreted as being specific to this sample rather than at the level of the entire MM population.

Contrary to what is documented in MCI and dementia, herein, MBI was non-dependent on sex, years of education, and marital status. The participants with MBI were younger than those of the non-MBI group (*p* = 0.009) (Table 2). The variability in the range of ages of participants included in the studies may be a reason for the reported mixed effects of age on MBI [22]. Herein, we included participants with a wider age range (60-87 years) than in other studies of MBI (i.e., participants of 72–79 years in Mortby et al., 2018) [17]; therefore, the association of age with MBI could be identified. Two research studies done with older adults who were cognitively unimpaired found that between 55% and 59% of individuals with emergent NPS convert to MCI [58,59]. In one of these studies, the group of patients with emergent NPS was younger than patients without NPS, and the clinically significant NPS were associated with a 3.92-fold increased risk of developing MCI [58]. Interestingly, in this study, being younger than <70 years doubled the risk of MBI compared to older participants.

In our sample, a subjective cognitive complaint and cognitive complaints reported by the informants were associated with an increased risk of MBI >4-fold and >15-fold, respectively (*p* < 0.001) (Table 4). Consequently, MBI is related to cognitive and functional decline [9,19] and is positioned as an early indicator of neurodegenerative disorders in which, as time progresses, behavioral symptoms are accompanied by cognitive symptoms [2,24,58,59,60]. Therefore, behavioral changes, which often present with cognitive complaints, might be early signs/symptoms of neurocognitive disorders [56].

We did not find differences in the MoCA total score between the groups according to the presence of MBI. This may be due to the fact that 69.12% of the older adults included were cognitively unimpaired (NC+SCD), since the correlation between MBI-C and MoCA total scores becomes stronger as cognitive impairment progresses [24].

SCD and MCI were associated with an increased risk for MBI (*p* < 0.05), in which the cognitive complaint, a common feature of both cognitive states, was also a risk factor for MBI. These findings support that MBI and cognitive status are interconnected conditions [22].

Depression assessed with BDI and depression history increased the risk of MBI five- and three-fold, respectively (*p* < 0.001) (Table 4). This agrees with previous studies that found an association between depressive symptoms or depression history and increased risk of MBI [22,61,62]. In this regard, the involvement of shared and bidirectional mechanisms between MBI and depression has been suggested [22]. In this context, clinicians should be aware of differentiating the symptoms of depression and anxiety from those MBI-associated symptoms, taking into account the age of onset and its evolution with cognitive decline [14].

Anxiety history and symptoms assessed with BAI increased the MBI risk two-fold and three-fold, respectively (Table 4). Anxiety is a risk factor for AD and vascular dementia [63]; in addition, MBI is related to AD biomarkers and greater volume of white matter hyperintensities, a sign of cerebrovascular disease [11,59,64,65]. Thus, the association between MBI and anxiety supports the hypothesis that this construct could be a clinical manifestation of these diseases.

A higher prevalence of two MBI subdomains was observed in *APOEε4/ε4* homozygotes: decreased motivation and abnormal perception or thought content. An association between *APOE* genotype and affective and emotional dysregulation has been previously documented; however, findings regarding the domain of decreased motivation are inconsistent [27,28]. The subdomain of decreased motivation has been associated with an increased risk of all-cause dementia in adults with NC over 55 years of age [16], suggesting a common biological substrate for these behavioral symptoms of pre-dementia stages with cognitive and functional aspects [22].

In a longitudinal study with 8.1 years of follow-up, the *APOEε4* carriers with emergent NPS had the highest rate of conversion to cognitive impairment compared to the other groups with a combination of presence/absence of NPS and *APOEε4* carrier status [59]. MBI is a multifactorial state that considers genetic predisposition (e.g., *APOEε4* allele) and other AD- and cerebrovascular disease-related factors [22,65]. Therefore, the risk association of MBI with age under 70 years and higher cognitive complaints, without a significant relationship with cognitive performance (MoCA score) identified in this study, could suggest that MBI is an even earlier clinical marker than cognitive impairment.

The variables accounting for the greatest variance in the dataset were CCQ, BDI, BAI, MBI-C, MoCA, age, and years of education. The first dimension of the PCA (PC1) predominantly clusters variables that measure affective–behavioral symptoms (neuropsychiatric axis) and cognitive complaints, while PC2 clusters variables that measure cognitive performance and associated sociodemographic variables (cognitive axis) (Figure 4B).

Additionally, a history of depression or anxiety, *APOEε4* carrier status, and hypertension were covariates significantly correlating in both PC1 and PC2. Our results indicate that these variables are common to both MCI and MBI, and therefore, they could be complementary syndromes [24,60]. Another approach is to consider MBI as a mediator state to explain the relationship between risk factors of brain health and cognitive decline [30].

There is evidence of an association between hypertension and an increased risk of MBI [22,30]. MBI, in turn, has been associated with a greater number of white matter hyperintensities in non-demented individuals (NC and MCI) from memory clinics [29,66]. It has been suggested that hypertension is a contributing factor to this imaging sign [67] and that both hypertension and white matter hyperintensities are markers of vascular cognitive impairment and dementia [67,68]. Thus, hypertension, a common condition in MBI and cognitive impairment, might have potential implications for cognitive or brain health aspects [30].

The strengths of the present integrative study are the inclusion of *APOE* genotype and other known AD risk factors in non-demented participants, the cognitive status, and their stratification by the presence of MBI. To our knowledge, this is the first report of MBI in MM older adults that analyzes its contribution to the variance of the dataset by using PCA.

We acknowledge a potential selection bias in our sample, in which 83.3% of the cohort were female participants. The main limitation is the low inclusion of male participants in our cohort (N = 41, 16.7%), limiting the generalizability of study findings; however, comparative analyses could be performed. In our experience, this corresponds to the inherent and typical characteristics of the participation of older adults in this type of study. Additionally, 1.6% of our participants were *APOE ε4/ε4* homozygotes, and therefore, our results of cognitive-MBI associations should be interpreted with caution since this is a cross-sectional study lacking detailed neuropsychological data. We recommend that multi-domain assessments be included in future longitudinal studies with similar populations, and that the number of participants homozygous for the *APOE*ε4 allele be increased according to its prevalence in the population of interest.

Our findings support the inclusion of the MBI in the protocols of assessment of non-demented older adults, as well as in combination with AD biomarkers and other neurodegenerative diseases [22,59,65]; in addition, it would be useful to improve risk stratification for older adults with cognitive complaints and symptoms of depression and/or anxiety. Longitudinal future research should delve deeper into: 1) establishing differences in MBI profile regarding risk of cognitive decline in MM population; 2) identifying MBI subtypes and determining whether their profiles are associated with the etiology of some neurodegenerative diseases; 3) evaluating MBI as an early detection marker of neurodegenerative diseases in the context of primary health care; 4) determining cognitive and imaging correlates of MBI; and 5) exploring MBI evolution and cognitive impairment, and their interactions with other factors.

## 5. Conclusions

The biplot of the first two principal components captured the maximum possible variance of the dataset: PC1 is a behavioral and affective component along with cognitive complaint, in which MBI had a significant loading, and PC2 corresponded to the cognitive dimension. Our association findings provide arguments in favor of including MBI in the assessment of non-demented MM elderly people.

## Figures and Tables

**Table 1:** Sociodemographic, cognitive, clinical, and genetic characteristics according to the cognitive status of the participants (N = 246).

	NC (N = 138)	SCD (N = 32)	MCI (N = 76)	*p*-Value
**Sex (%)** Male Female	22 (15.9) 116 (84.1)	4 (12.5) 28 (87.5)	15 (19.7) 61 (80.3)	0.616
**Age (in years)** μ ± SD Range	69.42 ± 6.27 60–87	69.4 ± 74.08 60–87	70.91 ± 6.40 60–85	0.277
**Y. education** μ ± SD Range	14.11 ± 3.22 6–22	14.05 ± 2.70 9–19	12.34 ± 4.19 6–22	0.004 **
**Marital status (%)** W/o partner With partner	81 (58.7) 57 (41.3)	23 (71.9) 9 (28.1)	40 (52.6) 36 (47.5)	0.179
**MoCA** μ ± SD Range	26.72 ± 1.65 25–30	26.94 ± 1.41 25–30	21.26 ± 2.31 12–24	≤0.001 *** SCD vs. MCI ≤ 0.001 *** NC vs. MCI ≤ 0.001 ***
**CCQ** μ ± SD Min-Max	10.04 ± 6.04 0–21	28.31 ± 5.99 22–43	18.07 ± 12.57 0–54	≤0.001 ***
**IQCODE** μ ± SD Range	81.67 ± 5.12 66–117	83.88 ± 9.79 40–101	86.70 ± 8.57 78–125	≤0.001 *** NC vs. SCD = 0.004 ** NC vs. MCI ≤ 0.001 ***
**BDI** μ ± SD Range	4.31 ± 4.31 0–22	8.22 ± 5.98 1–23	7.78 ± 6.50 0–25	≤0.001 *** NC vs. SCD ≤ 0.001 *** NC vs. MCI ≤ 0.001 ***
**BAI** μ ± SD Range	3.18 ± 4.19 0–23	8.09 ± 6.32 0–21	5.66 ± 5.95 0–29	≤0.001 *** NC vs. SCD ≤ 0.001 *** NC vs. MCI = 0.004 **
***APOE*** N (%) Allele *ε2* Allele *ε3* Allele *ε4*	5 (1.81) 250 (90.58) 21 (7.61)	2 (3.13) 51 (79.69) 11 (17.18)	3 (1.97) 129 (84.87) 20 (13.16)	0.793 NC vs. SCD&MCI = 0.021 * SCD vs. NC&MCI = 0.018 *

Kruskal–Wallis and post hoc tests were used for comparisons of three groups. Fisher (two groups) and χ^2^ (≥3 groups) tests were used for categorical variables, * *p* < 0.05, ** *p* < 0.01, and *** *p* < 0.001. NC, normal cognition; SCD, subjective cognitive decline; MCI, mild cognitive impairment; N, number; μ, mean value; SD, standard deviation; Y, years; MoCA, Montreal Cognitive Assessment; CCQ, Cognitive Complaint Questionnaire; IQCODE, Informant Questionnaire on Cognitive Decline in the Elderly; BDI, Beck Depression Inventory; BAI, Beck Anxiety Inventory.

**Table 2:** Comparison of sociodemographic, cognitive, and clinical characteristics, as well as *APOE* status, between participants with and without MBI.

Characteristic	Total (N = 246)	MBI		*p*-Value
		Yes (N = 91)	No (N = 155)	
**Cognitive status** (%) NC SCD MCI	138 32 76	36 (26.1) 19 (59.4) 36 (47.4)	102 (73.9) 13 (40.6) 40 (52.6)	≤0.001 *** 0.006 ** 0.032 *
**Sex** (%) Male Female	41 205	19(46.3) 72 (35.1)	22 (53.7) 133 (64.9)	0.215
**Age** (years)	μ ± SD Range	68.48 ± 6.42 60–84	70.70 ± 6.60 60–87	0.009 **
**Y. education**	μ ± SD Range	13.73 ± 3.79 6–22	13.46 ± 3.44 6–22	0.379
**Marital status** (%) With partner W/o partner	102 144	44 (48.4) 47 (51.6)	58 (37.4) 97 (62.6)	0.108
**Antecedents** (%) ^§^ Depression Anxiety Diabetes type 2 Hypertension COVID-19	100 70 61 106 139	49 (53.8) 33 (36.3) 22 (24.2) 46 (50.5) 56 (61.5)	51 (32.9) 37 (23.9) 39 (25.2) 60 (38.7) 83 (53.5)	0.002 ** 0.041 * 1.000 0.083 0.234
**Clinical scales** (μ ± SD) MoCA CCQ IQCODE BDI BAI	25.06 ± 3.14 14.89 ± 10.64 83.51 ± 7.38 5.89 ± 5.57 4.59 ± 5.37	24.85 ± 3.24 20.12 ± 11.77 88.56 ± 8.36 8.58 ± 6.36 6.70 ± 6.17	25.19 ± 3.09 11.83 ± 8.58 80.54 ± 4.66 4.31 ± 4.36 3.34 ± 4.40	0.440 ≤0.001 *** ≤0.001 *** ≤0.001 *** ≤0.001 ***
***APOE*** alleles (%) *ε2* *ε3* *ε4* *# copies of ε4 allele * 0 1 2	10 430 47 198 44 4	3 (1.65) 157 (86.26) 22 (12.09) 72 (79.1) 16 (17.6) 3 (3.3)	7 (2.26) 273 (88.06) 30(9.68) 126 (81.3) 28(18.1) 1 (0.67)	0.751 0.576 0.448 0.284

Data were analyzed using the Mann–Whitney test for continuous variables with 2 groups. Fisher and χ^2^ tests were used when 2 or ≥3 groups of categorical variables were compared, respectively. * *p* < 0.05, ** *p* < 0.01, and *** *p* < 0.001. NA, not applicable. N, number; μ, mean value; SD, standard deviation; Y, years; W/o, without; NC, normal cognition; SCD, subjective cognitive decline; MCI, mild cognitive impairment; MoCA, Montreal Cognitive Assessment; CCQ, Cognitive Complaint Questionnaire; IQCODE, Informant Questionnaire on Cognitive Decline in the Elderly; BDI, Beck Depression Inventory; BAI, Beck Anxiety Inventory. ^§^ Some participants reported ≥2 antecedents, and therefore, the percentages add up to >100. The confidence intervals of *APOE* allele frequencies for the group with MBI were as follows: (CI_95%_) *ε2* = 0.62–8.77, ε3 = 104.96–150.02, and *ε4* = 9.90–27.22. For the group without MBI, they were: (CI_95%_) *ε2* = 2.81–14.42, *ε3* = 232.17–296.78, and *ε4* = 20.24–42.83. The CIs for the MBI prevalence were: (CI_95%_) Total sample = 0.31–0.43, NC = 0.17–0.33, SCD = 0.42–0.76, and MCI = 0.36–0.58.

**Table 3:** Domains of the Mild Behavioral Impairment-Checklist (MBI-C) compared by *APOEε4* carrier status of participants.

MBI-C Scoring (μ ± SD)	Carriers of *APOEε4* Allele			Number of Copies of *APOEε4* Allele			
	Yes (N = 48)	No (N = 198)	*p*-Value	0 (N = 198)	1 (N = 44)	2 (N = 4)	*p*-Value
Total MBI-C	9.46 ± 12.58	7.21 ± 11.17	0.109	7.21 ± 11.17	8.55 ± 11.70	19.50 ± 19.28	0.114
Decreased motivation	1.75 ± 2.92	1.33 ± 2.92	0.129	1.33 ± 2.92	1.61 ± 2.80	3.25 ± 2.87	0.039 *^↟^
Affective and emotional dysregulation	2.50 ± 3.81	2.24 ± 3.67	0.716	2.24 ± 3.67	2.34 ± 3.74	4.25 ± 4.79	0.456
Impulse dyscontrol	4.21 ± 5.40	2.72 ± 4.06	0.054	2.72 ± 4.03	3.70 ± 4.85	9.75 ± 8.66	0.069
Social inappropriateness	0.56 ± 1.17	0.57 ± 1.24	0.848	0.57 ± 1.23	0.55 ± 1.19	0.75 ± 0.58	0.626
Abnormal perception or thought content	0.44 ± 1.54	0.36 ± 1.19	0.932	0.36 ± 1.19	0.34 ± 1.44	1.50 ± 2.38	0.063 ^↡^

Data were analyzed using the Mann–Whitney test or Kruskal–Wallis and post hoc test, accordingly. * *p* < 0.05. μ, mean value; SD, standard deviation. ^↟^ Comparison between 0 and 2 alleles, *p* = 0.013. ^↡^ Comparison between 0 and 2 alleles, *p* = 0.027 *, and 1 vs. 2, *p* = 0.021 *.

**Table 4:** Univariate analysis of association between variables of interest and presence of mild behavioral impairment.

Variable	Odds Ratio [CI_95%_]	*p*-Value
NC SCD	0.340 [0.199–0.581] 2.883 [1.348–6.166]	<0.001 *** 0.006 **
MCI Age ≥ 70 years Age < 70 years Subjective cognitive complaint (by CCQ) Informant cognitive complaint (by IQCODE)	1.882 [1.082–3.272] 0.550 [0.325–0.929] 1.819 [1.076–3.074] 4.706 [2.569–8.623] 15.889 [7.729–32.665]	0.025 * 0.025 * 0.025 * <0.001 *** <0.001 ***
Antecedent of depression	2.379 [1.399–4.047]	<0.001 ***
Depression (by BDI) Antecedent of anxiety	4.905 [2.595–9.271] 1.815 [1.032–3.192]	<0.001 *** 0.039 *
Anxiety (by BAI)	2.833 [1.625–4.939]	<0.001 ***

CI, confidence interval; NC, normal cognition; SCD, subjective cognitive decline; MCI, mild cognitive impairment; CCQ, Cognitive Complaint Questionnaire; IQCODE, Informant Questionnaire on Cognitive Decline in the Elderly; BDI, Beck Depression Inventory; BAI, Beck Anxiety Inventory. Cut-off values for BDI and BAI were 10 and 6, respectively. * *p* < 0.05, ** *p* < 0.01, and *** *p* < 0.001.

**Table 5:** Principal component analysis (PCA) outcomes.

Variable	Component		
	1	2	3
Eigenvalue	2.74	1.55	1.15
% Variance of the principal Components (PC)	34.3	19.3	14.4
% Contribution of variable (loadings) CCQ BDI MBI-C BAI IQCODE MoCA Years of education Age	20.276 (0.450) 20.146 (0.449) 20.113 (0.448) 18.293 (0.428) 15.323 (0.391) 3.800 (−0.195) 1.328 (−0.115) 0.719 (−0.085)	1.940 (0.139) 1.982 (0.141) 0.204 (−0.045) 2.865 (0.169) 2.337 (−0.153) 28.340 (0.532) 29.767 (0.546) 32.564 (−0.571)	1.359 (−0.117) 14.992 (−0.387) 19.506 (0.442) 20.181(−0.449) 35.246 (0.594) 0.313 (0.056) 3.173 (0.178) 5.231 (−0.229)

CCQ, Cognitive Complaint Questionnaire; BDI, Beck Depression Inventory; MBI-C, Mild Behavioral Impairment-Checklist; BAI, Beck Anxiety Inventory; IQCODE, Informant Questionnaire on Cognitive Decline in the Elderly; MoCA, Montreal Cognitive Assessment. A positive or negative value of the variable loading (directionality) indicates the type of correlation (same or opposite direction) with the component.

## Data Availability

The data presented in this study are available on request from the corresponding author due to confidentiality and ethical issues.

## Supplementary Materials

The following supporting information can be downloaded at: https://www.mdpi.com/article/10.3390/neurosci7020036/s1; Table S1: Comparison of MBI characteristics between the different cognitive statuses of the participants. Table S2: Comparison of sociodemographic, cognitive, and clinical characteristics, as well as *APOE* status, between male and female participants. Table S3: Comparison of the *APOEε4* allele frequencies observed in this work with those of previous studies carried out in Mexican-Mestizos [26,53,54,69]. Table S4: Domains of the Mild Behavioral Impairment-Checklist (MBI-C) compared with dominant and recessive models of *APOEε4* allele.

## Abbreviations

The following abbreviations are used in this manuscript:

AD	Alzheimer’s disease
APOE	Apolipoprotein E
BAI	Beck Anxiety Inventory
BDI	Beck Depression Inventory
CCQ	Cognitive Complaint Questionnaire
CTAD	Clinical Trials on Alzheimer’s Disease Conference
INAPAM	Instituto Nacional de las Personas Adultas Mayores
INNNMVS	Instituto Nacional de Neurología y Neurocirugía, Manuel Velasco Suárez
IQCODE	Informant Questionnaire on Cognitive Decline
ISTAART	International Society to Advance Alzheimer’s Research and Treatment
MBI	Mild behavioral impairment
MBI-C	Mild Behavioral Impairment Checklist
MCI	Mild cognitive impairment
MM	Mexican-Mestizos
MoCA	Montreal Cognitive Assessment
NC	Normal cognition
NPS	Neuropsychiatric symptoms
PCA	Principal Components Analysis
SCD	Subjective cognitive decline
SECIHTI	Secretaría de Ciencia, Humanidades, Tecnología e Innovación
SNV	Single-nucleotide variants
UAM	Universidad Autónoma Metropolitana
UNAM	Universidad Nacional Autónoma de México

## References

[1] Cummings J. (2021). The Role of Neuropsychiatric Symptoms in Research Diagnostic Criteria for Neurodegenerative Diseases. Am. J. Geriatr. Psychiatry.

[2] Ismail Z., Smith E.E., Geda Y., Sultzer D., Brodaty H., Smith G., Agüera-Ortiz L., Sweet R., Miller D., Lyketsos C.G. (2016). Neuropsychiatric symptoms as early manifestations of emergent dementia: Provisional diagnostic criteria for mild behavioral impairment. Alzheimer Dement..

[3] Soto M., Rosenberg P., Ballard C., Vellas B., Miller D., Gauthier S., Carrillo M.C., Lyketsos C., Ismail Z. (2024). CTAD Task Force Paper: Neuropsychiatric Symptoms in AD: Clinical Trials Targeting Mild Behavioral Impairment: A Report from the International CTAD Task Force. J. Prev. Alzheimers Dis..

[4] Camargo-Ortega E.A., Delgado-Martínez O., Martínez-Carrillo F., García-Peña C., Sosa-Ortiz A.L., Longoria-Ibarrola E.M., Ramírez-Bermúdez J., Ruiz-Garcia R. (2024). Are we missing detection of dementia at early stages in Mexico? A survey of dementia experts. Salud Publica Mex..

[5] Banning L.C.P., Ramakers I.H.G.B., Köhler S., Bron E.E., Verhey F.R.J., de Deyn P.P., Claassen J.A.H.R., Koek H.L., Middelkoop H.A.M., van der Flier W.M. (2020). The Association Between Biomarkers and Neuropsychiatric Symptoms Across the Alzheimer’s Disease Spectrum. Am. J. Geriatr. Psychiatry.

[6] Cozza M., Boccardi V. (2023). A narrative review on mild behavioural impairment: An exploration into its scientific perspectives. Aging Clin. Exp. Res..

[7] Chatzikostopoulos A., Moraitou D., Papaliagkas V., Tsolaki M. (2025). Mapping the Neuropsychiatric Symptoms in Alzheimer’s Disease Using Biomarkers, Cognitive Abilities, and Personality Traits: A Systematic Review. Diagnostics.

[8] Ferreira D.A., Macedo L.B.C., Foss M.P. (2024). Neuropsychiatric symptoms as a prodromal factor in Alzheimer’s type neurodegenerative disease: A scoping review. Clin. Neuropsychol..

[9] Ismail Z., McGirr A., Gill S., Hu S., Forkert N.D., Smith E.E. (2021). Mild Behavioral Impairment and Subjective Cognitive Decline Predict Cognitive and Functional Decline. J. Alzheimers Dis..

[10] Ismail Z., Agüera-Ortiz L., Brodaty H., Cieslak A., Cummings J., Fischer C.E., Gauthier S., Geda Y.E., Herrmann N., Kanji J. (2017). The Mild Behavioral Impairment Checklist (MBI-C): A Rating Scale for Neuropsychiatric Symptoms in Pre-Dementia Populations. J. Alzheimers Dis..

[11] Jin P., Xu J., Liao Z., Zhang Y., Wang Y., Sun W., Yu E. (2023). A review of current evidence for mild behavioral impairment as an early potential novel marker of Alzheimer’s disease. Front. Psychiatry.

[12] Guan D.X., Rehman T., Nathan S., Durrani R., Potvin O., Duchesne S., Pike G.B., Smith E.E., Ismail Z. (2024). Neuropsychiatric symptoms: Risk factor or disease marker? A study of structural imaging biomarkers of Alzheimer’s disease and incident cognitive decline. Hum. Brain Mapp..

[13] Ghahremani M., Smith E.E., Ismail Z. (2025). Improving dementia prognostication in cognitively normal older adults: Conventional versus novel approaches to modelling risk associated with neuropsychiatric symptoms. Br. J. Psychiatry.

[14] Ismail Z., Gatchel J., Bateman D.R., Barcelos-Ferreira R., Cantillon M., Jaeger J., Donovan N.J., Mortby M.E. (2018). Affective and emotional dysregulation as pre-dementia risk markers: Exploring the mild behavioral impairment symptoms of depression, anxiety, irritability, and euphoria. Int. Psychogeriatr..

[15] Creese B., Griffiths A., Brooker H., Corbett A., Aarsland D., Ballard C., Ismail Z. (2020). Profile of mild behavioral impairment and factor structure of the Mild Behavioral Impairment Checklist in cognitively normal older adults. Int. Psychogeriatr..

[16] Gracia-García P., López-Antón R., de la Cámara C., Santabárbara J., Lobo E., Lobo A. (2024). Mild behavioral impairment in the general population aged 55+ and its association with incident dementia. Alzheimers Dement..

[17] Mortby M.E., Ismail Z., Anstey K.J. (2018). Prevalence estimates of mild behavioral impairment in a population-based sample of pre-dementia states and cognitively healthy older adults. Int. Psychogeriatr..

[18] Pan Y., Shea Y.F., Li S., Chen R., Mak H.K., Chiu P.K., Chu L.W., Song Y.Q. (2021). Prevalence of mild behavioural impairment: A systematic review and meta-analysis. Psychogeriatrics.

[19] Park J.I., Alzheimer′s Disease Neuroimaging Initiative (2024). Prevalence of mild behavioural impairment and its association with cognitive and functional impairment in normal cognition, mild cognitive impairment, and mild Alzheimer’s dementia. Psychogeriatrics.

[20] Blasutto B., Fattapposta F., Casagrande M. (2025). Mild Behavioral Impairment and cognitive functions: A systematic review and meta-analysis. Ageing Res. Rev..

[21] Rouse H.J., Ismail Z., Andel R., Molinari V.A., Schinka J.A., Small B.J. (2024). Impact of Mild Behavioral Impairment on Longitudinal Changes in Cognition. J. Gerontol. A Biol. Sci. Med. Sci..

[22] Tang S.L., Subramaniam P., Siau C.S., Chong A.S.S., Liu F. (2025). Risk factors of mild behavioral impairment: A systematic review. Front. Psychol..

[23] Matuskova V., Veverova K., Jester D.J., Matoska V., Ismail Z., Sheardova K., Horakova H., Cerman J., Laczó J., Andel R. (2024). Mild behavioral impairment in early Alzheimer’s disease and its association with *APOE* and *BDNF* risk genetic polymorphisms. Alzheimers Res. Ther..

[24] Scheuermann J.S., Graessel E., Kratzer A., Scheerbaum P. (2024). Mild behavioral impairment in people with mild cognitive impairment: Are the two conditions related?. J. Alzheimers Dis..

[25] Jackson R.J., Hyman B.T., Serrano-Pozo A. (2024). Multifaceted roles of *APOE* in Alzheimer disease. Nat. Rev. Neurol..

[26] Genis-Mendoza A.D., Martínez-Magaña J.J., Bojórquez C., Téllez-Martínez J.A., Jiménez-Genchi J., Roche A., Bojorge A., Chávez M., Castañeda C., Guzmán R. (2018). Programa de detección del alelo *APOE-E4* en adultos mayores mexicanos con deterioro cognitivo. Gac. Med. Mex..

[27] Angelopoulou E., Koros C., Hatzimanolis A., Stefanis L., Scarmeas N., Papageorgiou S.G. (2024). Exploring the Genetic Landscape of Mild Behavioral Impairment as an Early Marker of Cognitive Decline: An Updated Review Focusing on Alzheimer’s Disease. Int. J. Mol. Sci..

[28] Vellone D., Ghahremani M., Goodarzi Z., Forkert N.D., Smith E.E., Ismail Z. (2022). Apathy and *APOE* in mild behavioral impairment, and risk for incident dementia. Alzheimers Dement..

[29] Suriyawong W., Sanprakhon P., Pipatpiboon N., Chaiwong N., Budda R., Thaipisuttikul P. (2025). Mild Behavioral Impairment as a Mediator of the Relationships Among Perceived Stress, Social Support, Physical Activity, and Cognitive Function in Older Adults with Transitional Cognitive Decline: A Structural Equation Modelling Analysis. Can. J. Aging.

[30] Guan D.X., Aundhakar A., Tomaszewski Farias S., Ballard C., Creese B., Corbett A., Pickering E., Roach P., Smith E.E., Ismail Z. (2025). Vascular risk factor associations with subjective cognitive decline and mild behavioral impairment. Brain Commun..

[31] Creese B., Arathimos R., Brooker H., Aarsland D., Corbett A., Lewis C., Ballard C., Ismail Z. (2021). Genetic risk for Alzheimer’s disease, cognition, and mild behavioral impairment in healthy older adults. Alzheimers Dement..

[32] Cruz-Orduña I., Bellón J.M., Torrero P., Aparicio E., Sanz A., Mula N., Marzana G., Begué C., Cabezón D., Olazarán J. (2012). Detecting MCI and dementia in primary care: Effectiveness of the MMS, the FAQ and the IQCODE. Fam. Pract..

[33] Nuñez S.L., Bruno D. (2021). Validación del Cuestionario de Quejas Cognitivas. Neurol. Argent..

[34] Nasreddine Z.S., Phillips N.A., Bédirian V., Charbonneau S., Whitehead V., Collin I., Cummings J.L., Chertkow H. (2005). The Montreal Cognitive Assessment, MoCA: A Brief Screening Tool for Mild Cognitive Impairment. J. Am. Geriatr. Soc..

[35] Aguilar-Navarro S.G., Mimenza-Alvarado A.J., Palacios-García A.A., Samudio-Cruz A., Gutiérrez-Gutiérrez L.A., Ávila-Funes J.A. (2018). Validity and Reliability of the Spanish Version of the Montreal Cognitive Assessment (MoCA) for the Detection of Cognitive Impairment in Mexico. Rev. Colomb. Psiquiatr..

[36] Islam N., Hashem R., Gad M., Brown A., Levis B., Renoux C., Thombs B.D., McInnes M.D. (2023). Accuracy of the Montreal Cognitive Assessment tool for detecting mild cognitive impairment: A systematic review and meta-analysis. Alzheimers Dement..

[37] Pugh E.A., Kemp E.C., van Dyck C.H., Mecca A.P., Sharp E.S. (2018). Effects of Normative Adjustments to the Montreal Cognitive Assessment. Am. J. Geriatr. Psychiatry.

[38] Parra-Rodríguez L., Silva-Pereyra J., Sánchez-García S., García-Peña C., Flores-Vázquez J.F., Roa-Rojas P. (2025). Normative Data for the Montreal Cognitive Assessment (MoCA) in Mexican Adults: A Regression-Based Approach. Diagnostics.

[39] Villegas M.E., Méndez L., Rodríguez F., Loperena V., Varela R. (1998). La estandarización del Inventario de Depresión de Beck para los residentes de la Ciudad de México. Salud Ment..

[40] Robles R., Varela R., Jurado S., Páez F. (2001). Versión mexicana del Inventario de Ansiedad de Beck: Propiedades psicométricas. Rev. Mex. Psicol..

[41] Del-Ser T., Morales J.M., Barquero M.S., Cantón R., Bermejo F. (1997). Application of a Spanish version of the “Informant Questionnaire on Cognitive Decline in the Elderly” in the clinical assessment of dementia. Alzheimer Dis. Assoc. Disord..

[42] Aguera-Ortiz L.F., Lopez-Alvarez J., Del Nido-Varo L., Soria Garcia-Rosel E., Perez-Martinez D.A., Ismail Z. (2017). Mild behavioural impairment as an antecedent of dementia: Presentation of the diagnostic criteria and the Spanish version of the MBI-C scale for its evaluation. Rev. Neurol..

[43] Cui Y., Dai S., Miao Z., Zhong Y., Liu Y., Liu L., Jing D., Bai Y., Kong Y., Sun W. (2019). Reliability and Validity of the Chinese Version of the Mild Behavioral Impairment Checklist for Screening for Alzheimer’s Disease. J. Alzheimers Dis..

[44] Matsuoka T., Ismail Z., Imai A., Shibata K., Nakamura K., Nishimura Y., Rubinstein E., Uchida H., Mimura M., Narumoto J. (2024). Relationship between Loneliness and Mild Behavioral Impairment: Validation of the Japanese Version of the MBI Checklist and a Cross-Sectional Study. J. Alzheimers Dis..

[45] Jack C.R., Andrews J.S., Beach T.G., Buracchio T., Dunn B., Graf A., Hansson O., Ho C., Jagust W., McDade E. (2024). Revised criteria for diagnosis and staging of Alzheimer’s disease: Alzheimer’s Association Workgroup. Alzheimers Dement..

[46] Jack C.R., Bennett D.A., Blennow K., Carrillo M.C., Dunn B., Haeberlein S.B., Holtzman D.M., Jagust W., Jessen F., Karlawish J. (2018). NIA-AA Research Framework: Toward a biological definition of Alzheimer’s disease. Alzheimers Dement..

[47] Jessen F., Amariglio R.E., Buckley R.F., van der Flier W.M., Han Y., Molinuevo J.L., Rabin L., Rentz D.M., Rodriguez-Gomez O., Saykin A.J. (2020). The characterisation of subjective cognitive decline. Lancet Neurol..

[48] Petersen R.C. (2016). Mild Cognitive Impairment. Continuum.

[49] Winblad B., Palmer K., Kivipelto M., Jelic V., Fratiglioni L., Wahlund L.O., Nordberg A., Bäckman L., Albert M., Almkvist O. (2004). Mild cognitive impairment-beyond controversies, towards a consensus: Report of the International Working Group on Mild Cognitive Impairment. J. Intern. Med..

[50] Greenacre M., Groenen P.J.F., Hastie T., Iodice D’Enza A., Markos A., Tuzhilina H. (2022). Principal Component Analysis. Rev. Methods Primers.

[51] Olivoto T., Dal’Col Lúcio A. (2020). metan: An R package for multi-environment trial analysis. Methods Ecol. Evol..

[52] Barberena-Jonas C., Flores-Ocampo V., Ogonowski N.S., Piña-Escudero S.D., Mata I.F., Yokoyama J.S., García-García L., Aguilar Salinas C.A., Tusié-Luna M.T., Moreno-Estrada A. (2025). Genetic analysis of *APOE* reveals distinct origins and distribution of ancestry-enrichment haplotypes in the Mexican Biobank. Genes Dis..

[53] López M., Guerrero J., Yescas P., Boll M.C., Familiar I., Ochoa A., Rasmussen A., Alonso M.E. (2007). Apolipoprotein E ε4 allele is associated with Parkinson disease risk in a Mexican Mestizo population. Mov. Disord..

[54] Martínez-Magaña J.J., Genis-Mendoza A.D., Tovilla-Zarate C.A., González-Castro T.B., Juárez-Rojo I.E., Hernández-Díaz Y., Martinez-Hernandez A.G., García-Ortiz H., Orozco L., López-Narvaez M.L. (2019). Association between *APOE* polymorphisms and lipid profile in Mexican Amerindian population. Mol. Genet. Genom. Med..

[55] Sheikh F., Ismail Z., Mortby M.E., Barber P., Cieslak A., Fischer K., Granger R., Hogan D.B., Mackie A., Maxwell C.J. (2018). Prevalence of mild behavioral impairment in mild cognitive impairment and subjective cognitive decline, and its association with caregiver burden. Int. Psychogeriatr..

[56] Leow Y.J., Soo S.A., Kumar D., Zailan F.Z.B., Sandhu G.K., Vipin A., Lee F.P.H.E., Ghildiyal S., Liew S.Y., Dang C. (2024). Mild Behavioral Impairment and Cerebrovascular Profiles Are Associated with Early Cognitive Impairment in a Community-Based Southeast Asian Cohort. J. Alzheimers Dis..

[57] Pan Y., Shea Y.F., Ismail Z., Mak H.K., Chiu P.K., Chu L.W., Song Y.Q. (2022). Prevalence of mild behavioural impairment domains: A meta-analysis. Psychogeriatrics.

[58] Wise E.A., Rosenberg P.B., Lyketsos C.G., Leoutsakos J.M. (2019). Time course of neuropsychiatric symptoms and cognitive diagnosis in National Alzheimer’s Coordinating Centers volunteers. Alzheimers Dement..

[59] Kim T.H., Head E., Stark C.E.L., Sordo L., Kirby K.A., Corona M.G., McDonnell M., Grill J.D., Sultzer D.L. (2025). Late-life emergence of neuropsychiatric symptoms and risk of cognitive impairment in cognitively unimpaired individuals. Alzheimers Dement..

[60] Mallo S.C., Ismail Z., Pereiro A.X., Facal D., Lojo-Seoane C., Campos-Magdaleno M., Juncos-Rabadán O. (2019). Assessing mild behavioral impairment with the mild behavioral impairment checklist in people with subjective cognitive decline. Int. Psychogeriatr..

[61] Taragano F.E., Allegri R.F., Krupitzki H., Sarasola D.R., Serrano C.M., Loñ L., Lyketsos C.G. (2009). Mild behavioral impairment and risk of dementia: A prospective cohort study of 358 patients. J. Clin. Psychiatry.

[62] Rao A.R., Chatterjee P., Thakral M., Dwivedi S.N., Dey A.B. (2020). Behavioural issues in late life may be the precursor of dementia- A cross sectional evidence from memory clinic of AIIMS, India. PLoS ONE.

[63] Becker E., Orellana Rios C.L., Lahmann C., Rücker G., Bauer J., Boeker M. (2018). Anxiety as a risk factor of Alzheimer’s disease and vascular dementia. Br. J. Psychiatry.

[64] Creese B., Ismail Z. (2022). Mild behavioral impairment: Measurement and clinical correlates of a novel marker of preclinical Alzheimer’s disease. Alzheimers Res. Ther..

[65] Kan C.N., Hilal S., Xu X., Venketasubramanian N., Chen C., Tan C.H. (2025). Comorbid cerebrovascular and neurodegenerative burden in mild behavioural impairment and their impact on clinical trajectory. Acta Neuropsychiatr..

[66] Miao R., Chen H.Y., Robert P., Smith E.E., Ismail Z., MEMENTO Study Group (2021). White matter hyperintensities and mild behavioral impairment: Findings from the MEMENTO cohort study. Cereb. Circ. Cogn. Behav..

[67] Wardlaw J.M., Smith C., Dichgans M. (2019). Small vessel disease: Mechanisms and clinical implications. Lancet Neurol..

[68] Livingston G., Huntley J., Liu K.Y., Costafreda S.G., Selbæk G., Alladi S., Ames D., Banerjee S., Burns A., Brayne C. (2024). Dementia prevention, intervention, and care: 2024 report of the Lancet standing Commission. Lancet.

[69] Lozano-Tovar S., Rodríguez-Agudelo Y., Dávila-Ortiz de Montellano D.J., Pérez-Aldana B.E., Ortega-Vázquez A., Monroy-Jaramillo N. (2023). Relationship between APOE, PER2, PER3 and OX2R Genetic Variants and Neuropsychiatric Symptoms in Patients with Alzheimer’s Disease. Int. J. Environ. Res. Public. Health.

