# Supplementary material for: First Analysis of Mild Behavioral Impairment in a Sample of Mexican Older Adults
Source: NeuroSci. 2026 Mar 13;7(2):36. doi: 10.3390/neurosci7020036 (PMC13010659; doi:10.3390/neurosci7020036)
Supplement: Supplementary file 1 [file neurosci-07-00036-s001.zip › neurosci-4129759-supplementary.pdf]

Supplementary Material

Table S1. Comparison of MBI characteristics between the different cognitive status of the participants.

	**NC (N=138)**	**SCD (N=32)**	**MCI (N=76)**	***p*-value**
**MBI-C** μ ±SD				
Total	4.92±8.94	11.47±14.24	11.00±12.99	≤0.001***<br>NC vs SCD≤0.001***<br>NC vs MCI≤0.001***
Decreased motivation	0.99±2.57	2.22±3.56	1.86±3.05	≤0.001***<br>NC vs SCD=0.010*<br>NC vs MCI=0.008**
Affective and emotional dysregulation	1.49±3.09	3.38±4.54	3.29±4.00	≤0.001***<br>NC vs SCD=0.012*<br>NC vs MCI≤0.001***
Impulse dyscontrol	1.86±3.12	4.56±5.30	4.43±5.28	≤0.001***<br>NC vs SCD≤0.001***<br>NC vs MCI=0.002**
Social inappropriateness	0.38±0.94	0.66±1.26	0.87±1.60	0.014*<br>NC vs MCI=0.005**
Abnormal perception or thought content	0.21 ±0.92	0.66±1.56	0.55 ±1.60	0.013*<br>NC vs SCD=0.004*<br>NC vs MCI=0.046*

Statistical significance levels: *p<0.05, **p<0.01 and p<0.001*** obtained by Kruskal-Wallis Test with post-hoc test. NC, normal cognition; SCD, subjective cognitive decline; MCI, mild cognitive impairment; N, number; M, mean value; SD, standard deviation; MBI-C, Mild Behavioral Impairment Checklist.

Table S2. Comparison of sociodemographic, cognitive, and clinical characteristics, as well as *APOE* status between male and female participants.

Characteristic	Sex		*p*-value
	**Male (N=41)**	**Female (N=205)**	
**Cognitive status** (%)			
NC	22 (53.6)	116 (56.6)	
SCD	4 (9.8)	28 (13.6)	0.616
MCI	15 (36.6)	61 (29.8)	
**Age** (years)			
μ ±SD	70.88±7.32	69.68±6.43	0.372
Range	60 – 87	60 – 87	
**Y. education**			
μ ±SD	15.02±3.27	13.26±3.57	0.004**
Range	6 – 22	6 – 22	
**Marital status** (%)			
With partner	31 (76.5)	71 (34.6)	≤0.001***
W/o partner	10 (24.4)	134 (65.4)	
**Antecedents** (%)§			
Depression	8 (19.5)	92 (44.9)	0.003**
Anxiety	7 (17.1)	63 (30.7)	0.089
Diabetes type 2	8 (19.5)	33 (25.9)	0.436
Hypertension	24 (58.5)	82 (40.0)	0.038*
COVID-19	23 (56.1)	116 (56.6)	1.00
**Clinical scales** (μ±SD)			
MoCA	24.32±2.91	25.21±3.17	0.067
CCQ	11.83±8.46	15.51±10.94	0.065
IQCODE	83.59±6.89	83.49±7.48	0.995
BDI	4.46±4.91	6.18±5.66	0.039*
BAI	3.29±5.16	4.84±5.38	0.022
**MBI-C**			
Total	9.73±12.12	7.23±11.32	0.131
Decreased motivation	2.07±3.51	1.28±2.75	0.079
Affective and emotional dysregulation	1.85±2.76	2.38±3.86	0.924
Impulse dyscontrol	4.46±5.24	2.72±4.15	0.052
Social inappropriateness	0.93±1.63	0.49±1.11	0.124
Abnormal perception or thought content	0.41±1.30	0.37±1.26	0.966
**# copies of *APOEε4* allele** (%)			
0	32 (78.0)	166 (81.0)	0.196
1	7 (17.1)	37 (18.0)	
2	2 (4.9)	2 (1.0)	

Statistical significance levels: *p<0.05, **p<0.01 and p<0.001*** obtained by Mann-Whitney U Test. NC, normal cognition; SCD, subjective cognitive decline; MCI, mild cognitive impairment; N, number; M, mean value; SD, standard deviation; MBI-C, Mild Behavioral Impairment Checklist.

Table S3. Comparison of the *APOEε4* allele frequencies observed in this work with those of previous studies carried out in Mexican-Mestizos.

Population	N	*APOEε4* allele frequency (n)	*p*-value
Mexican Mestizo controls and patients with Parkinson disease [53]	229	0.079 (18)	0.356
Mexican Amerindian population [54]	1997	0.107 (214)	1.000
Mexican Mestizo controls and patients with Alzheimer's Disease [69]	31	0.08 (5)	0.375
Mexican older adults with cognitive decline [26]	1307	0.101 (105)	0.222
This study	246	0.106 (26)	—

Comparisons were obtained by Fisher's Exact Test. N, number.

Table S4. Domains of the Mild Behavioral Impairment-Checklist (MBI-C) compared by dominant and recessive models of *APOEε4* allele.

MBI-C scoring (μ±SD)	*APOEε4* dominant model			*APOEε4* recessive model		
	Ht + HmA (N=48)	Hm (N=198)	*p*-value	Hm+Ht (N=242)	HmA (N=4)	*p*-value
Total	9.46±12.59	7.21±11.17	0.109	7.45±11.26	19.50±19.28	0.088
Decreased motivation	1.75±2.82	1.33±2.92	0.129	1.38±2.91	3.25±2.87	0.017*
Affective and emotional dysregulation	2.50±3.81	2.24±3.67	0.716	2.26±3.68	4.25±4.79	0.211
Impulse dyscontrol	4.21±5.40	2.72±4.06	0.054	2.90±4.22	9.75±8.66	0.082
Social inappropriateness	0.56±1.17	0.57±1.24	0.848	0.56±1.23	0.75±0.96	0.334
Abnormal perception or thought content	0.44±1.54	0.36±1.19	0.932	0.36±1.24	1.50±2.38	0.022*

Statistical significance level: *$p<0.05$ obtained by Mann-Whitney U Test. N, number; M, mean value; SD, standard deviation; MBI-C, Mild Behavioral Impairment Checklist; Hm, homozygous for the reference *APOEε4* allele; Ht, heterozygous; HmA, Homozygous for the alternative *APOEε4* allele.
